# Responsiveness and sensitivity of PROMs to change in disease activity status in early and established rheumatoid arthritis

**DOI:** 10.1093/rheumatology/keae213

**Published:** 2024-04-04

**Authors:** Agnes E M Looijen, Elise van Mulligen, Harald E Vonkeman, Annette H M van der Helm-van Mil, Pascal H P de Jong

**Affiliations:** Department of Rheumatology, Erasmus MC, Rotterdam, The Netherlands; Department of Rheumatology, Erasmus MC, Rotterdam, The Netherlands; Department of Rheumatology, Leiden University Medical Centre, Leiden, The Netherlands; Department of Rheumatology and Clinical Immunology, Medisch Spectrum Twente, Enschede, The Netherlands; Department of Psychology, Health and Technology, University of Twente, Enschede, The Netherlands; Department of Rheumatology, Erasmus MC, Rotterdam, The Netherlands; Department of Rheumatology, Leiden University Medical Centre, Leiden, The Netherlands; Department of Rheumatology, Erasmus MC, Rotterdam, The Netherlands

**Keywords:** remote monitoring, patient reported outcomes, disease activity status, rheumatoid arthritis

## Abstract

**Objectives:**

To determine whether patient-reported outcome measures (PROMs) capturing activity limitations, health impact, pain, fatigue and work ability are responsive and sensitive to changes in disease activity status in patients with early and established RA.

**Methods:**

All early RA patients (*n* = 557) from the tREACH trial and established RA patients (*n* = 188) from the TARA trial were included. Both studies were multicentre, single-blinded trials with a treat-to-target management approach. The following PROMs were studied: HAQ Disability Index (HAQ-DI), morning stiffness severity, EQ-5D, general health, 36-item short form (SF-36), joint pain, fatigue and productivity loss. Mean changes in PROMs between two consecutive visits were compared with changes in disease activity status (remission, low disease activity and active disease) using linear mixed models and standardized response means. Additionally, the proportion of individual observations that showed an expected PROM response to disease activity status alterations was calculated.

**Results:**

HAQ-DI, morning stiffness severity, general health, EQ-5D and joint pain demonstrated responsiveness to improvement or worsening of disease activity status in both early and established RA. SF-36 physical and mental component scale, fatigue and productivity loss did not show this effect in both groups. Across nearly all PROMs, the magnitude of change and the proportion of individual observations that reflect a shift from and to active disease remained low.

**Conclusion:**

HAQ-DI, morning stiffness severity, EQ-5D, general health and joint pain are responsive to disease activity status alterations on a group level in both early and established RA. For the individual patient the responsiveness of these PROMs is poor.

**Clinical trial registration:**

tREACH trial (www.isrctn.com, ISRCTN26791028) and TARA trial (www.onderzoekmetmensen.nl, NTR2754)

Rheumatology key messages

- Patient-reported outcome measures (PROMs) covering functionality, health impact and pain are responsive to disease activity status alterations on a population level.
- For individual observations, the responsiveness of one single PROM remains poor.
- Due to poor responsiveness in individual observations, individual PROMs are unsuitable for remote monitoring purposes.

## Introduction

Thanks to the currently recommended treat-to-target approach, the majority of RA patients achieve well-controlled disease [1–4]. Within this approach, intensification and tapering of treatment is based on the disease activity status of the patient, which is derived from the DAS. RA patients can be classified into the following disease activity status, which are based on the DAS thresholds: (i) remission; (ii) low disease activity; and (iii) moderate to active disease [1]. Regular joint examinations by a trained healthcare professional are essential for accurately assessing this disease activity status and determining the need for treatment intensification or tapering [1, 2, 4]. However, to maintain this high standard of care, despite increasing demands on healthcare provision, it is desirable to assess a patient’s health status through remote monitoring as an addition to routine outpatient clinic follow-up [5–7].

Remote monitoring is already implemented in the management of several chronic diseases such as diabetes, hypertension and congestive heart failure [7, 8]. In the field of rheumatology, remote monitoring has also been acknowledged for its potential benefits. It may, for example, lead to improved disease control by enabling early flare detection, as well as lowering the frequency of visits in the outpatient clinic [7]. Remote monitoring could be facilitated through measurement of patient-reported outcome measures (PROMs). One major advantage of using PROMs for remote monitoring is the capability to capture them with digital applications that can automatically calculate PROM scores [7].

A systematic review including various studies on digitally collected PROMs suggested that PROMs can be used for remote monitoring and that this approach does not seem to be inferior to regular care [9]. However, most of the included studies in this review used a combination of interventions which means that the evidence for the use of PROMs alone for monitoring disease activity is limited. Subsequently, studies on remote monitoring often measure PROMs without directly reporting whether the results of the PROM scores reflect the patient’s disease activity status [9]. As the patient’s and physician’s perspective on disease activity do not always align, there lies a challenge in the use of PROMs that adequately reflect a patients disease activity status [10]. Some literature has shown that functionality, joint pain, morning stiffness, general health, health-related quality of life and fatigue change with the development and resolution of a disease flare [11]. Other studies on the other hand have shown that some of these outcomes, especially fatigue, have a weak correlation with disease activity [12].

Outcome domains that are recommended by the International Consortium for Health-Outcome Measurement (ICHOM) for patients with inflammatory arthritis are: activity limitations, health impact, pain, fatigue and work ability [13]. PROMs that capture these domains are increasingly implemented in daily practice, as healthcare is shifting towards patient-centered care. The responsiveness and sensitivity to change of PROMs covering aforementioned domains have mainly been investigated by comparing them with a disease activity index on a continuous scale, or over the course of a therapeutic intervention with known effectiveness [14–21]. Despite the informative nature of these data, they provide limited insight into the responsiveness of PROMs in detecting changes in disease activity status, which is needed for our treat-to-target approach.

We hypothesize that ICHOM-recommended PROMs that correlate well with inflammation, including PROMs on functionality health impact and pain, also change with disease activity status over time and are therefore usable for remote monitoring alongside their use for patient-centered care [11, 14–16]. Therefore, our aim is to determine whether PROMs that cover the ICHOM-recommended domains are responsive and sensitive to disease activity status alterations in patients with early as well as established RA.

## Methods

### Patients

For this study we selected all patients who participated in the ‘treatment in the Rotterdam Early Arthritis Cohort’ trial (tREACH, ISRCTN26791028) and ‘Tapering strategies in Rheumatoid Arthritis’ trial (TARA, NTR2754). Patients were included if they had their disease activity measured at minimally two consecutive visits and had one or more PROM score at those two consecutive visits. The included patients in the tREACH trial have early RA or undifferentiated arthritis (UA) and the patients in the TARA trial have established RA. RA diagnosis was based upon whether patients met the 1987 or 2010 classification criteria [22, 23]. The medical ethics committee of Erasmus MC approved both the tREACH trial (MEC-2006-252) and TARA trial (MEC-2011-141). All medical ethics committees of each participating centre approved the study protocols for local feasibility. All patients provided written informed consent prior to inclusion.

### Study design

The tREACH trial was a multicentre, stratified, single-blinded, randomized controlled trial with a treat-to-target approach that compared different initial treatment strategies. This trial has been described in detail elsewhere [24]. DMARD-naïve early RA and UA patients with one or more swollen joint were included. Patients received either (i) MTX, including DMARD combination therapies with or without glucocorticoid bridging therapy; (ii) HCQ; or (iii) NSAIDs/glucocorticoids as initial treatment. Treatment was aimed at reaching low disease activity (LDA) and intensified in case of active disease, defined as DAS > 2.4. Medication was tapered when patients were in sustained remission, defined as DAS < 1.6 at two consecutive visits. If a flare occurred, defined as a DAS > 2.4, full treatment was restarted according to stage of the protocol.

The TARA trial was a multicentre, single-blinded, randomized controlled trial that compared tapering of a conventional synthetic DMARD with a TNF-inhibitor (TNFi) in established RA patients. This trial has been described in detail elsewhere [25]. Established RA patients with a well-controlled disease, defined as DAS ≤ 2.4 and swollen joint count ≤ 1, using one or more conventional synthetic DMARD and TNFi were included. Participants were randomized into two groups which gradually tapered their conventional DMARD first followed by the TNFi, or vice versa. When a disease flare occurred, defined as DAS > 2.4 or swollen joint count > 1, the last effective treatment was restarted and intensified until well-controlled disease was re-established.

### Data collection

In both the tREACH and TARA trials, visits took place every 3 months for 36 and 24 months, respectively. At each visit the DAS was measured and PROMs were collected. In our analyses we included PROMs that cover the following ICHOM-recommended outcome domains: activity limitations, health impact, pain, fatigue and work ability [13]. Table 1 shows the collected PROMs with the time intervals between measurements and their minimal clinically important differences, if known. Supplementary Table S1, available at *Rheumatology* online, shows the exact questions that were asked for the single-item PROMs, which includes morning stiffness, general health/patient global assessment (PGA), pain, fatigue and presenteeism. For the multi-item PROMs, i.e. the 3-level EQ-5D (EQ-5D-3L), the validated Dutch versions were used.

**Table 1.** Included PROMs per outcome domain stratified for early and established RA

			Time-interval of collection (months)	
Outcome domain	Used PROM	MCID	Early RA	Established RA
Activity limitations	HAQ-DI [26–28]	≥0.22	3	3
	Morning stiffness severity (NRS 0–10)	n/a	n/a	3
Health impact	EQ-5D-3L [29–31]	≥0.04	3	3
	General health/PGA^b^ (VAS 0–100mm) [32]	≥10	3	3
	SF-36 [32–34] PCS and MCS	≥2.5–5	3/6^a^	6
Pain	Joint pain (NRS/SRS 0–10) [35, 36]	≥1	3	3
Fatigue	Fatigue (VAS 0–100mm) [32]	≥10	3/6^a^	3
Work ability	Productivity loss (presenteeism, 0–100%)	n/a	3	3

a During follow-up questionnaires were assessed either every 3 months or 6 months.

b For the early RA cohort, the VAS general health was assessed. For the established RA cohort, the patient global assessment was assessed. EQ-5D-3L: 3-level EQ-5D; HAQ-DI: HAQ Disability Index; MCID: minimal clinically important difference; MCS: mental component score; n/a: not applicable; NRS: numeric rating scale; PCS: physical component score; PGA: patient global assessment; PROM: patient-reported outcome measure; SF-36: RAND 36-item short form health survey; SRS: semantic rating scale; VAS: visual analogue scale.

#### Activity limitations

In both groups, activity limitation was measured with the HAQ Disability Index (HAQ-DI) [26, 27]. The cumulative score ranges from 0 to 3, and higher scores indicate more functional impairment. In established RA, severity of morning stiffness was also assessed using a numeric rating scale (NRS) ranging from 0 to 10. Higher scores correspond with more severe experienced morning stiffness.

#### Health impact

Health impact was measured with (i) EQ-5D-3L, (ii) RAND 36-item short form health survey (SF-36) and (iii) general health or PGA. The EQ-5D-3L questionnaire measures health related quality of life [29, 30]. It contains five health domains which are answered using a 3-point Likert scale varying from no problems to extreme problems. The answers are converted into utility scores based on Dutch reference values [37]. Scores range from 0 to 1; 0 equals death and 1 equals perfect health. The SF-36 contains of 36 questions covering the following eight domains: physical functioning, physical role limitations, bodily pain, general health, vitality, emotional role limitations, social functioning and mental health [33, 34]. These domains are summarized into a physical component summary score (PCS) and a mental component summary score (MCS). Lastly, general health was measured in the early RA cohort, and a PGA was measured in established RA. Both general health and PGA were assessed using a visual analogue scale (VAS) of 0–100 mm. Higher scores indicate a worse experienced general health or PGA.

#### Pain and fatigue

Joint pain was measured on a semantic rating scale ranging from 0 to 10 in early RA and an NRS of 0–10 in established RA. Higher scores indicate more experienced joint pain. Fatigue was measured with a VAS from 0 to 100 mm in early RA, while an NRS ranging from 0 to 10 was used in established RA. For comparability, this NRS was transformed to a 0–100 scale. Higher scores indicate more experienced fatigue.

#### Work ability

For this study we included presenteeism as measure for work ability. Presenteeism is defined as loss of productivity due to working while sick. It was assessed with an NRS ranging from 0 to 10. Higher scores indicate less productivity loss. These scores were transformed into percentages ranging from 0% to 100%; 0% indicates no productivity loss while 100% represents no productivity at all.

### Statistical analysis

Responsiveness was defined as the ability of a PROM to detect a disease activity status alteration [38, 39]. Sensitivity to change was defined as the ability to detect any change and the magnitude of this change [38, 39]. On a population level, we measured the mean responsiveness as well as sensitivity to change. We also assessed responsiveness on a patient level by assessing the proportion of individual observations with expected changes in PROM score when altering from either remission or LDA to active disease and vice versa.

Changes in disease activity status between two consecutive visits were compared with changes in PROMs to assess responsiveness on a population level. Disease activity statuses were defined as: (i) active disease (DAS > 2.4), (ii) LDA (DAS ≤ 2.4 and ≥1.6) and (iii) remission (DAS < 1.6) [40]. Multiple changes in disease activity status were assessed within one patient. This resulted in a maximum of 12 observations over time for early RA and 8 observations over time for established RA, respectively. Mean changes in PROMs per disease activity status alteration between two consecutive visits were compared with stable disease using linear mixed models. The models were fitted with an autoregressive structure for the residual errors and restricted maximum likelihood estimation. Analyses were stratified for early and established RA. In the linear mixed models, we corrected for repeated measurements within the patients and initial treatment or tapering strategy.

The magnitude of change of PROMs over time for the different disease activity status alterations was evaluated with the standardized response mean (SRM). The SRM is calculated by dividing the mean difference in PROM score between two time points by the s.d. of its difference [41]. The changes in PROMs were considered as trivial (SRM <0.2), small (0.2 ≤ SRM < 0.5), moderate (0.5 ≤ SRM < 0.8) or large (SRM ≥0.8) [39, 42]. To avoid over- or underestimation of this effect size due to correlation between subsequent visits, we corrected for the correlations between the repeated measurements as proposed by Middel *et al.* [41]. The used formula is shown in Supplementary Fig. S1, available at *Rheumatology* online.

To understand the responsiveness of PROMs on an individual level, we also assessed the proportion of observations with an expected PROM change when the disease activity status changed from well-controlled disease (DAS ≤ 2.4) to active disease (DAS > 2.4) or vice versa. In case of deterioration or improvement of disease activity status, PROMs are expected to deteriorate or improve accordingly. The percentage of observations with the expected change in PROM score was calculated.

Lastly, two sensitivity analyses were performed. To exclude observations that change from disease activity status with a minimal change in DAS, we performed aforementioned analyses only including observations with a minimal DAS change of >0.6 [43]. We also analysed responsiveness and sensitivity to change in PROMs to disease activity status alterations using the 3-item DAS, i.e. the DAS without general health or PGA included. We added this analysis, to assess if the responsiveness and sensitivity to change alters when the patient-reported component (general health or PGA) was removed, because studies have shown that this component can remain elevated even in the absence of inflammation [44, 45].

To adjust for multiple testing a Bonferroni correction was applied. The calculated *P*-values were corrected through multiplication by 18, which is the number of performed tests. *P*-values ≤0.05 were considered statistically significant. Analyses were performed using Stata version 18 (StataCorp, College Station, TX, USA).

## Results

### Patient characteristics of our early and established RA cohort

Baseline characteristics of the 557 early and 188 established RA patients are shown in Table 2. The median symptom duration (interquartile range) was 0.4 (0.3–0.6) years for early RA and 6.2 (4.1–8.9) years for established RA. The mean (s.d.) DAS at baseline was 3.1 (1.0) in the early and 1.0 (0.5) in the established RA group. As the early RA cohort also included UA patients, ACPA and RF positivity differs between both cohorts: 40% of the early RA patients were ACPA and RF positive, compared with 72% and 68% of the established RA patients. Age and sex were comparable between both groups.

**Table 2.** Baseline characteristics for early and established RA

	Early RA (*n* = 557)	Established RA (*n* = 188)
Sex, female, *n* (%)	373 (67)	124 (66)
Age (years), mean (s.d.)	52 (14)	57 (12)
ACPA positivity, *n* (%)	222 (40)	134 (72)
RF positivity, *n* (%)	223 (40)	127 (68)
Symptom duration (years)	0.4 (0.3–0.6)	6.2 (4.1–8.9)
Disease activity score, mean (s.d.)	3.1 (1.0)	1.0 (0.5)
44-swollen joint count	6 (3–10)	0 (0–0)
53-tender joint count	8 (3–13)	0 (0–1)
ESR (mm/h)	19 (11–35)	8 (3–15)
VAS general health/PGA	50 (30–66)	14 (4–27)

All results are shown as median (interquartile range) unless indicated otherwise. *n*: number; PGA: patient global assessment; VAS: visual analogue scale.

### Mean responsiveness of PROMs to improvement or worsening of disease activity status

**Figure 1.**
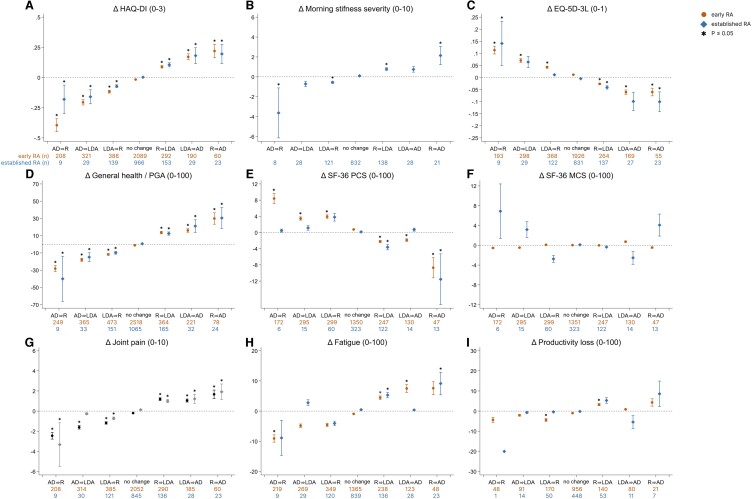
Mean change in PROM score per change in disease activity status. (**A**–**I**) The mean change (95% CI) in PROM per disease activity status alteration for early and established RA patients. ^*^Indicates that the change in PROM score is significantly (*P* ≤ 0.05) different compared with stable disease activity status after correction for multiple testing. AD: active disease; EQ-5D-3L: 3-level EQ-5D; HAQ-DI: HAQ Disability Index; LDA: low disease activity; MCS: mental component score; NRS: numeric rating scale; PCS: physical component score; PGA: patient global assessment; PROM: patient-reported outcome measure; R: remission; SF-36: RAND 36-item short form health survey; VAS: visual analogue scale

Figure 1 shows the mean change (95% CI and significance) in PROM scores per change in disease activity status. In both early and established RA, HAQ-DI, morning stiffness severity, EQ-5D, general health/PGA and joint pain were responsive to improvement or worsening of the disease activity status (Fig. 1A–D and G). For example, the HAQ-DI score increases with deterioration of the disease activity status and decreases when the disease activity status improves. In most of these PROMs, changes from and to active disease had a comparable or bigger impact on the change in PROM score compared with changes between LDA and remission. SF-36 PCS, fatigue and productivity loss showed responsiveness to change in early RA, but not clearly in established RA (Fig. 1E, H and I). SF-36 MCS showed no responsiveness with changes in disease activity status for both early as well as established RA (Fig. 1F).

Of the responsive PROMs, general health/PGA and HAQ-DI showed significant differences between all disease activity status alterations and stable disease in both early and established RA (Fig. 1A and D). Morning stiffness severity, EQ-5D and joint pain mostly showed significant differences in early RA and for some disease activity alterations in established RA (Fig. 1B, C and G). For SF-36 PCS fatigue and productivity loss, significant differences with stable disease were mostly seen in disease activity status alterations in early RA (Fig. 1E, H and I). The mean change in PROM score most often exceeded the minimal clinically important difference in disease activity status alterations from or to active disease.

### Sensitivity to change per disease activity status alteration

Table 3 shows the SRM per PROM and disease activity status alteration for early and established RA combined. Responsive PROMs, i.e. HAQ-DI, morning stiffness, EQ-5D, general health/PGA and joint pain, had SRMs ranging from mostly trivial and small (i.e. HAQ-DI) to moderate and large (i.e. general health/PGA). Moderate to large SRMs were predominantly seen in disease activity status alterations from remission to active disease and vice versa. SF-36 MCS, fatigue and productivity loss, showed the lowest SRMs.

**Table 3.** Corrected standardized response means per PROM score and disease status alteration

	AD⇒R	AD⇒LDA	LDA⇒R	R⇒LDA	LDA⇒AD	R⇒AD
HAQ-DI	0.68	0.38	0.19	0.18	0.33	0.36
Morning stiffness severity	1.21	0.32	0.23	0.33	0.35	0.78
EQ-5D-3L	0.63	0.34	0.24	0.22	0.33	0.42
General health/PGA	1.52	0.90	0.62	0.72	0.88	1.46
SF36 PCS	0.84	0.35	0.40	0.25	0.17	1.01
SF36 MCS	0.03	0.03	0.04	0.01	0.04	0.05
Joint pain	1.01	0.59	0.45	0.48	0.42	0.68
Fatigue	0.34	0.17	0.17	0.19	0.25	0.31
Productivity loss	0.24	0.09	0.22	0.22	0.01	0.29

AD: active disease; EQ-5D-3L: 3-level EQ-5D; HAQ-DI: HAQ Disability Index; LDA: low disease activity; MCS: mental component score; PCS: physical component score; PGA: patient global assessment; PROM: patient-reported outcome measure; R: remission; SF-36: RAND 36-item short form health survey.

### Responsiveness in individual observations

**Figure 2.**
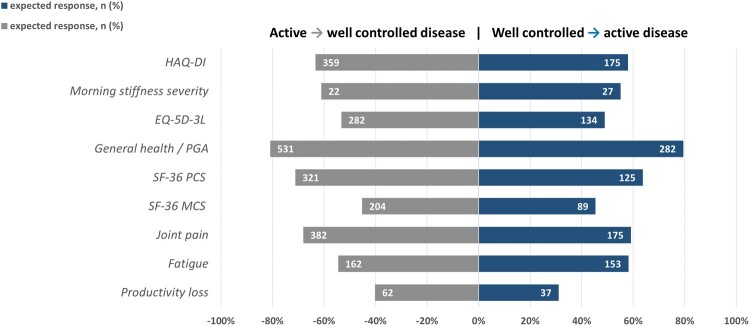
Proportion of observations with an expected change in PROM score. The proportion of RA observations, early as well as established, with expected changes in PROM score when changing from active (DAS >2.4) to well-controlled disease (DAS ≤2.4) and vice versa. EQ-5D-3L: 3-level EQ-5D; HAQ-DI: HAQ Disability Index; MCID: minimal clinically important difference; MCS: mental component score; PCS: physical component score; PGA: patient global assessment; PROM: patient-reported outcome measure; SF-36: RAND 36-item short form health survey

Figure 2 shows the proportion of all RA observations, early as well as established, with expected changes in PROMs when changing from active (DAS > 2.4) to well-controlled disease (DAS ≤ 2.4) and vice versa. Expected changes in PROMs due to a change from active disease to well-controlled disease, or vice versa, were most often seen in general health/PGA (81% and 79%, respectively), SF-36 PCS (71% and 64%, respectively) and joint pain (68% and 59%, respectively). For the responsive PROMs the proportion of observations with an expected response ranged from 49% to 81%. SF36 MCS and productivity loss showed the least observations with an expected response.

### Sensitivity analyses

Both sensitivity analyses showed similar results with regard to which PROMs are responsive to disease activity status alterations as well as the sensitivity to change per disease activity status alteration (Supplementary Figs S2–S4 and Table S2, available at *Rheumatology* online.). If only observations with a DAS change of >0.6 were included, the responsiveness and sensitivity to change increased on a population, as well as an individual level. When the 3-item DAS (i.e. the DAS without general health or PGA) was used, the responsiveness and sensitivity to change decreased on a population level as well as for the individual patient.

## Discussion

In this study we investigated whether PROMs that cover the recommended ICHOM outcome domains are responsive and sensitive to changes in disease activity status. In our study, HAQ-DI, morning stiffness severity, EQ-5D, general health/PGA and joint pain were responsive to improvement or worsening of disease activity status in early as well as established RA patients. SF-36 PCS, fatigue and productivity loss showed a trend in early RA. In contrast, the SF-36 MCS did not show any changes that were concordant with disease activity status alterations. In some responsive PROMs, for example EQ-5D, HAQ-DI and general health and PGA, changes were bigger when patients switched to and from active disease compared with changes between LDA and remission. Corrected SRMs were moderate to low for most PROMs, except for general health and PGA. The proportion of observations with an expected response when changing from and to active disease varied between 49% and 81% in the responsive PROMs.

These results are in line with the current literature which suggests that PROMs in the domains of functionality, health impact and pain change together with disease activity or with improvement after treatment [13, 16–18]. This study adds to other studies by addressing responsiveness and sensitivity of PROMs in relation to changes in disease activity status. In addition, most previous studies only investigated the responsiveness or sensitivity of PROMs in relation to an effective treatment intervention, which often only leads to improvement in disease activity [14–17, 19–21]. However, for remote monitoring it is of utmost importance that all clinically relevant changes, including disease flares, can be detected adequately as this requires a change in treatment according to the currently recommend treat-to-target management approach [1, 2, 4, 7].

Our findings may give guidance to future innovations in remote monitoring as they show which PROMs might be valuable to use. Although some PROMs show responsiveness to disease activity status alterations on a group level, the magnitude of this change as well as the responsiveness of one single PROM for the individual patient is poor. This suggests that there is not one specific PROM that is useful enough for remote monitoring. As an alternative, a combination of several responsive PROMs might give a better insight to disease activity status alterations. Therefore, future research should focus on combining different responsive PROMs to improve its discriminative ability for the individual patient. To the best of our knowledge, only one study found an association between a combination of several PROMs and a disease flare, with a sensitivity of ∼56% and specificity of ∼70%, which may leave room for improvement [46].

In addition, other factors should also be taken into account to improve the feasibility and reliability of remote monitoring. Among others, the application should be easy to use for both patients and physicians [47]. In order to minimize questionnaire burden, the frequency as well as the number of items asked should also be taken into account [47]. To minimize the number of items, computer-adaptive tests could be used [48]. Future research could also focus on combining a selection of the most sensitive items of each PROM into one condensed outcome measure. Moreover, to enhance reliability, PROMs could also be combined with laboratory values and (digital) biomarkers such as (passive) smartphone usage and wearable devices [49, 50].

Strengths of our study include usage of both early and established RA patients from two randomized controlled trials with a treat-to-target management approach and fixed medication protocol. Moreover, the data include assessment of PROMs that cover the recommended ICHOM domains over several years of follow-up [13].

Limitations include the small number of observations for some PROMs, especially in the switch from remission to active disease or vice versa. This could be explained by the treat-to-target approach in both trials and low number of disease flares and it could potentially result in underestimation of the effect size or significance of the results. Furthermore, patients who became lost to follow-up or incompletely filled out questionnaires could have led to biased results. However, when assessing the pattern of missingness between different disease activity statuses, the overall dropout seems to be evenly distributed. Analysing several PROMs over time has led to multiple testing and repeated measurements. We statistically addressed these issues by correcting for multiple testing using a Bonferroni correction and a linear mixed model that takes repeated measurements into account. In addition, it is important to note that the single-item PROM questions such as fatigue, pain and productivity loss were similar but not the same between both cohorts. Moreover, not all of these PROMs were derived from validated questionnaires, therefore validation of these results is necessary.

In conclusion, HAQ-DI, morning stiffness severity, EQ-5D, general health/PGA and joint pain are responsive to disease activity status alterations on a group level in both early as well as established RA. However, for the individual patient the responsiveness of one of these single responsive PROMs is poor. Although, aforementioned PROMs may be helpful in remote monitoring of patients with RA. A combination of PROMs, with or without other clinical outcomes, may improve overall discriminative ability.

## Supplementary Material

keae213_Supplementary_Data

## Data Availability

The authors confirm that the data supporting the findings of this study are available within the article and its supplementary materials, available at *Rheumatology* online.
