# Supplementary material for: Responsiveness and sensitivity of PROMs to change in disease activity status in early and established rheumatoid arthritis
Source: Rheumatology (Oxford). 2024 Apr 4;64(3):1060–7. doi: 10.1093/rheumatology/keae213 (PMC11879326; doi:10.1093/rheumatology/keae213)
Supplement: keae213_Supplementary_Data [file keae213_supplementary_data.pdf]

Supplementary material

Responsiveness and sensitivity of PROMs to change in disease activity status in early and established rheumatoid arthritis

Supplementary table S1. One-item PROMs, including their anchors

**Morning stiffness severity**		
**A. Established RA**	**Morning stiffness severity**	
Scale	Numeric rating scale 0 – 10	
Question[1]	Kleur het vakje in dat het beste de ernst van uw ochtendstijfheid beschrijft gedurende de afgelopen 7 dagen.	Color the box that best describes the severity of your morning stiffness over the past 7 days.
Lower anchor	Geen	None
Upper anchor	Extreem	Extreme
Source	Exact source unknown	
**Health impact**		
**B. Early RA**	**General health (early RA)**	
Scale	Visual analogue scale 0-100 millimeters	
Question	Zet nu een streepje op de lijn dat volgens u aangeeft hoe goed of hoe slecht u uw gezondheidstoestand van de afgelopen week waardeert als u daarbij denkt aan uw algehele (lichamelijke, geestelijke en sociale) gezondheid.	Now indicate on the line how well or how poorly you rate your state of health over the past week considering your overall (physical, mental and social) health.
Lower anchor	Beste gezondheidstoestand	Best health state
Upper anchor	Slechtste gezondheidstoestand	Worst health state
Source	Exact source unknown	
**C. Established RA**	**Patient global assessment**	
Question	Als u denkt aan alle manieren waarop de gewrichtsontstekingen u hebben beïnvloed. Hoe is het dan vandaag met uw gewrichtsontstekingen gesteld?	When you think of all the ways arthritis has affected you. How is your arthritis doing today?
Lower anchor	Heel goed	Very good
Upper anchor	Heel slecht	Very bad
Source	Exact source unknown	
**Pain**		
**D. Early RA**	**Joint pain**	
Scale	Semantic rating scale 0 – 10	
Question	Hoeveel gewrichtspijn heeft u vandaag?	How severe is your arthritis pain today?
Lower anchor	Helemaal geen pijn	No pain
Upper anchor	Heel erg veel pijn	Unbearable pain
Source	Rheumatoid Arthritis Disease Activity Index(1)	
**E. Established RA**	**Joint pain**	
Scale	Numeric rating scale 0 – 10	
Question	Geef het cijfer dat het beste de pijn beschrijft die u door uw reumatoïde artritis (RA) ervaart gedurende de afgelopen 7 dagen.	Indicate the number that best describes the pain you experienced due to your rheumatoid arthritis (RA) over the past 7 days.
Lower anchor	Geen	None
Upper anchor	Extreem	Extreme
Source	Exact source unknown	
**Fatigue**		
**F. Early RA**	**Fatigue**	
Scale	Visual analogue scale 0 – 100 millimetres	
Question	Zet een streepje op de lijn dat volgens u aangeeft hoe u uw vermoeidheid van de afgelopen week waardeert.	Indicate on the line how you rate your fatigue over the past week
Lower anchor	Helemaal niet moe	Not tired at all
Upper anchor	Heel erg moe	Very tired
Source	Exact source unknown	
**G. Established RA**	**Fatigue**	
Scale	Numeric rating scale 0 – 10	
Question	Geef het cijfer aan dat de gemiddelde mate van vermoeidheid gedurende de afgelopen 7 dagen weergeeft.	Indicate the number which shows the average level of fatigue during the past 7 days.
Lower anchor	Geen vermoeidheid	No fatigue
Upper anchor	Totaal uitgeput	Totally exhaused
Source	Bristol Rheumatoid Arthritis Fatigue Multi-Dimensional Questionnaire(2)	

[1]Questions were translated in English in case that no English version of the question was directly available. In case of an multilingual version, the English question was reported.

Abbreviations: PROM, patient-reported outcome measure; RA, Rheumatoid Arthritis.

Supplementary table S1. Continued Included one-item PROM-questions including their anchors

Work ability		
**H. Early RA**	**Presenteeism**	
Scale	Numeric rating scale 1 – 10	
Question	Wilt u aangeven hoe goed u hebt gewerkt op de dagen dat u wel op uw werk was terwijl u last had van gezondheidsproblemen?	Please indicate how well you worked on the days you did attend work while suffering from health problems?
Lower anchor	Zeer slecht	Very bad
Upper anchor	Heel goed	Very good
Source	The Health and Labour questionnaire(3)	
**I. Established RA**	**Presenteeism**	
Scale	Numeric rating scale 0 – 10	
Question	Op de dagen dat u last had, kon u misschien niet zoveel werk doen als normaal. Hoeveel werk kon u op deze dagen gemiddeld doen?	On the days when you were suffering from problems, perhaps you were not able to do as much work as normal. On those days, how much work could you do on average?
Lower anchor	Ik kon op deze dagen niets doen	I was not able to do anything on those days
Upper anchor	Ik kon net zoveel doen als normaal	I was able to do just as much as normal.
Source	iMTA Productivity Cost Questionnaire(4)	

[1]Questions were translated in English in case that no English version of the question was directly available. In case of an multilingual version, the English question was reported.

Abbreviations: PROM, patient-reported outcome measure; RA, Rheumatoid Arthritis;

Supplementary figure S1. SRM corrected for correlation between two repeated longitudinal measurements (5)

$$SRM_{corrected} = \frac{\bar{X}1 - \bar{X}2}{SD_{\bar{X}1-\bar{X}2}} * \sqrt{2} * \sqrt{1-r}$$

Abbreviations: $\bar{X}$, *overall mean score in patient-reported outcome measure; r, correlation between two longitudinal measurements; and SRM, standardised response mean;*

Supplementary figure S2. Mean change in PROM-score per change in disease activity status and a minimal DAS change of >0.6

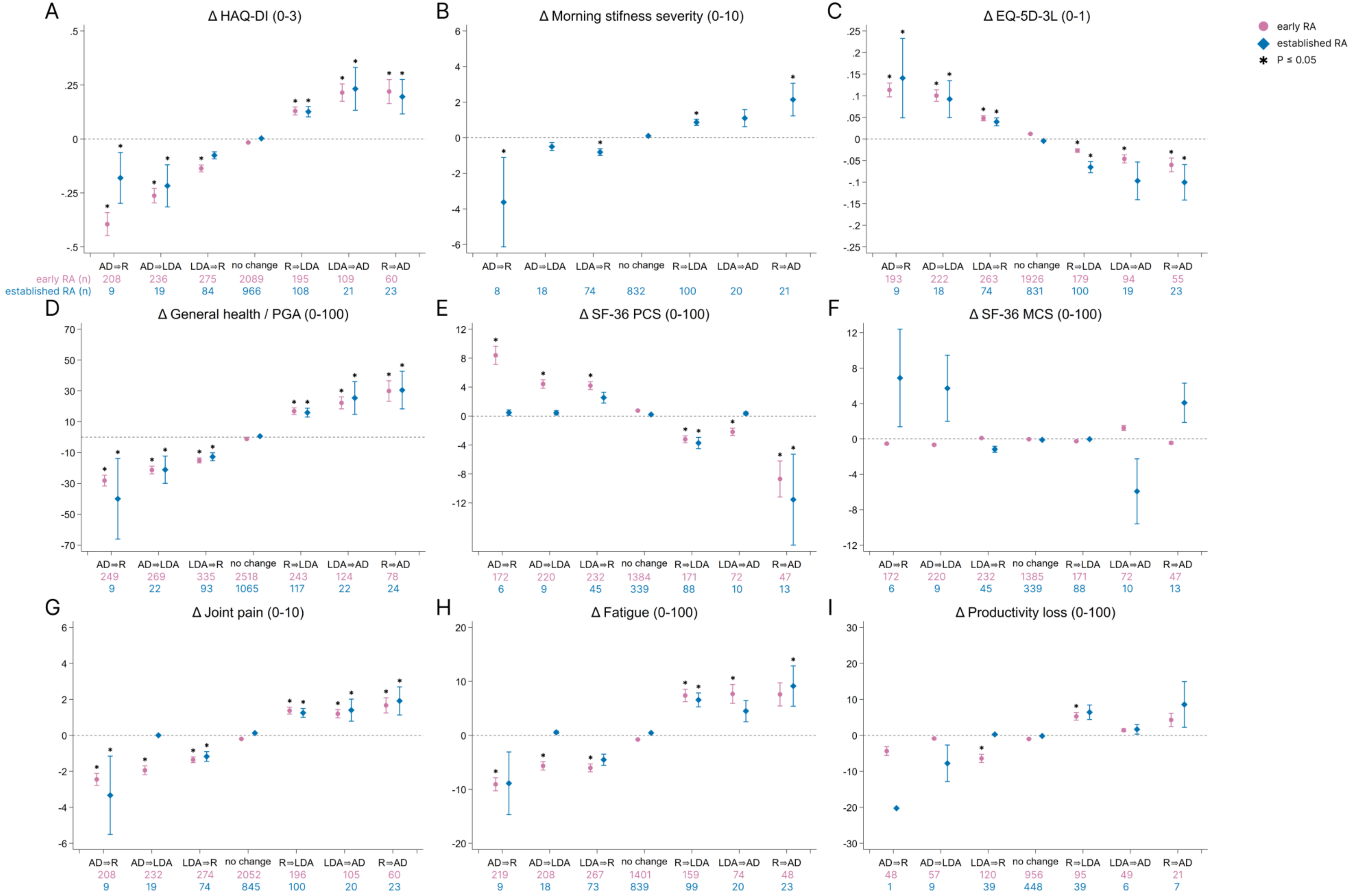

Figure S2A-I show the mean change (95%CI) in PROM-score per disease activity status alteration for early and established RA patients. This figure only includes observations for which the shift in disease status is accompanied with a minimal change in DAS of >0.6.

* indicates that the change in PROM is significantly different from observations without a disease activity status alteration, including correction for multiple testing (p<0.05).

Abbreviations: AD, active disease; EQ-5D-3L, 3-level EQ-5D; HAQ-DI, Health Assessment Questionnaire Disability Index; LDA, low disease activity; MCS, mental component score; NRS, numeric rating scale; PCS, physical component score; PGA, patient global assessment; PROM, patient-reported outcome measure; R, remission; SF-36, RAND 36-item short form health survey; and VAS, visual analogue scale.

Supplementary figure S3. Mean change in PROM-score per change in disease activity status with the 3-item DAS as disease activity measure

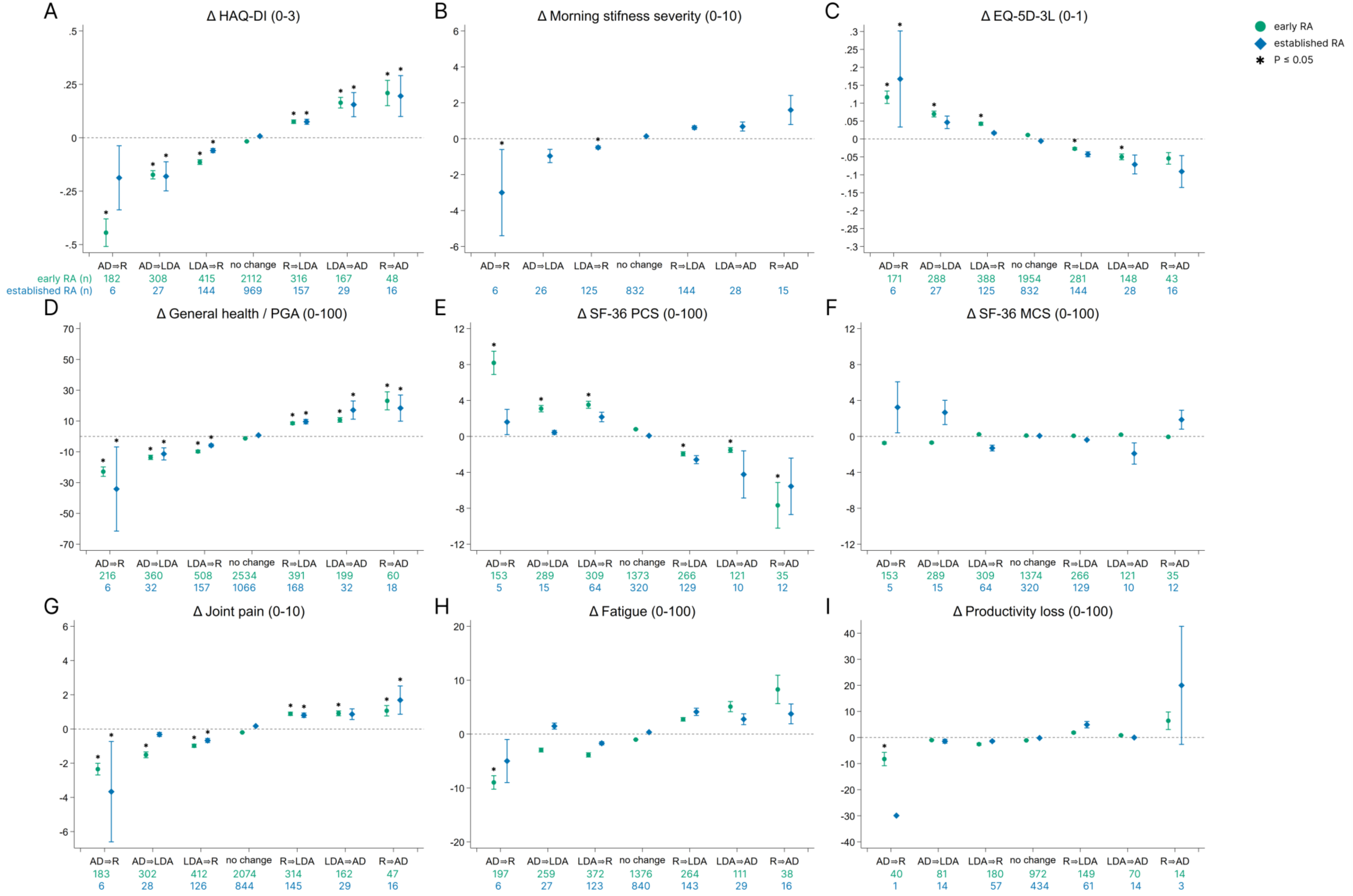

Figure S3 shows the mean change (95%CI) in PROM-score per disease activity status alteration for early and established RA patients. This figure shows results of disease status alterations calculated with the 3-item DAS as disease activity measure.

* indicates that the change in PROM-score is significantly different from patients without a disease activity status alterations, including correction for multiple testing (p<0.05).

Abbreviations: AD, active disease; EQ-5D-3L, 3-level EQ-5D; HAQ-DI, Health Assessment Questionnaire Disability Index; LDA, low disease activity; MCS, mental component score; NRS, numeric rating scale; PCS, physical component score; PGA, patient global assessment; PROM, patient-reported outcome measure; R, remission; SF-36, RAND 36-item short form health survey; and VAS, visual analogue scale.

Supplementary table S2. Corrected standardized response means per PROM-score and disease status alteration, using (A) observations with a minimal DAS change of >0.6 and (B) 3-item DAS as disease activity measure.

A

	AD⇒R	AD⇒LDA	LDA⇒R	R⇒LDA	LDA⇒AD	R⇒AD
HAQ-DI	0,69	0,48	0,23	0,25	0,41	0,36
Morning stifness severity	1,21	0,20	0,33	0,38	0,48	0,78
EQ-5D-3L	0,63	0,48	0,32	0,29	0,27	0,42
General health / PGA	1,52	1,11	0,81	0,90	1,27	1,46
SF36 PCS	0,84	0,45	0,40	0,32	0,19	1,01
SF36 MCS	0,03	0,04	0,01	0,02	0,03	0,05
Joint pain	1,01	0,72	0,55	0,57	0,49	0,68
Fatigue	0,34	0,21	0,22	0,28	0,27	0,30
Productivity loss	0,18	0,12	0,32	0,29	0,13	0,33

trivial change (SRM<0.2)
small change (SRM≥0.2, <0.5)
moderate change (SRM≥0.5, <0.8)
large change (SRM>0.8)

B

	AD⇒R	AD⇒LDA	LDA⇒R	R⇒LDA	LDA⇒AD	R⇒AD
HAQ-DI	0,77	0,31	0,18	0,14	0,31	0,33
Morning stifness severity	0,95	0,41	0,19	0,25	0,30	0,59
EQ-5D-3L	0,62	0,34	0,23	0,21	0,29	0,37
General health / PGA	1,17	0,63	0,45	0,44	0,55	1,00
SF36 PCS	0,82	0,30	0,33	0,20	0,19	0,68
SF36 MCS	0,06	0,05	0,00	0,01	0,00	0,04
Joint pain	0,94	0,54	0,37	0,35	0,35	0,48
Fatigue	0,33	0,10	0,13	0,12	0,18	0,28
Productivity loss	0,38	0,05	0,14	0,16	0,04	0,43

trivial change (SRM<0.2)
small change (SRM≥0.2, <0.5)
moderate change (SRM≥0.5, <0.8)
large change (SRM>0.8)

Abbreviations: AD, active disease; EQ-5D-3L, 3-level EQ-5D; HAQ-DI, Health Assessment Questionnaire Disability Index; LDA, low disease activity MCS, mental component score; PCS, physical component score; PGA, patient global assessment; PROM, patient-reported outcome measure; R, remission; and SF-36, RAND 36-item short form health survey.

Supplementary figure S4. Proportion of observations with an expected change in PROM-score using (A) observations with a minimal DAS change of >0.6 and (B) 3-item DAS as disease activity measure

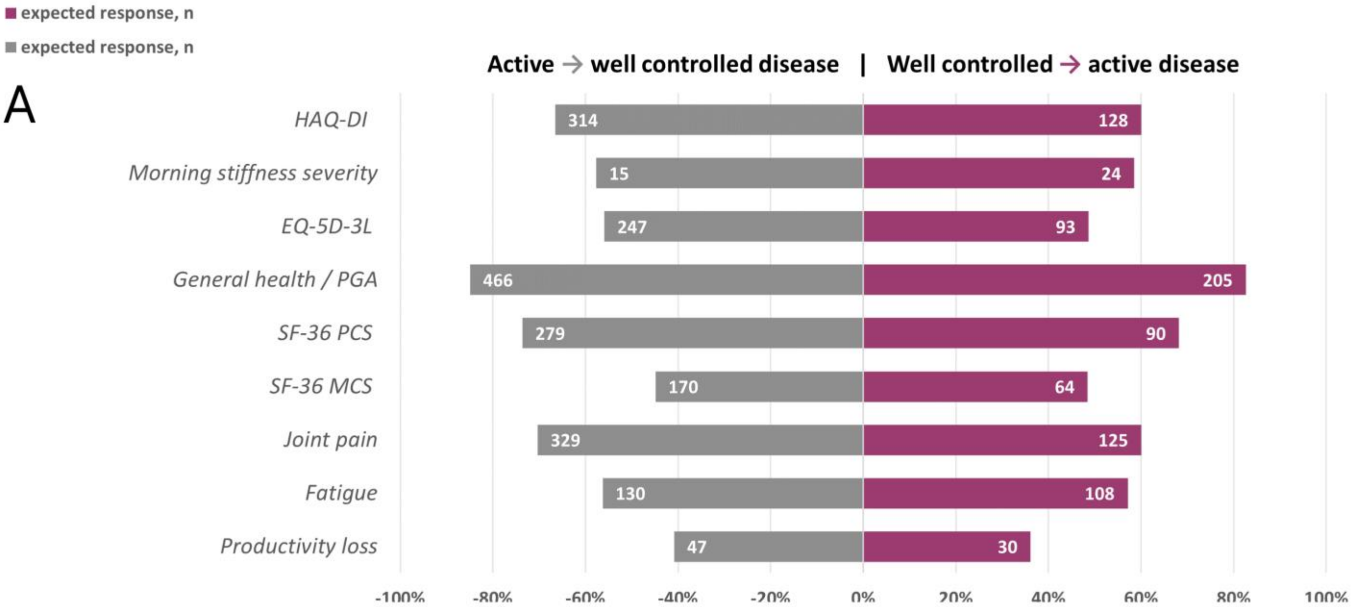

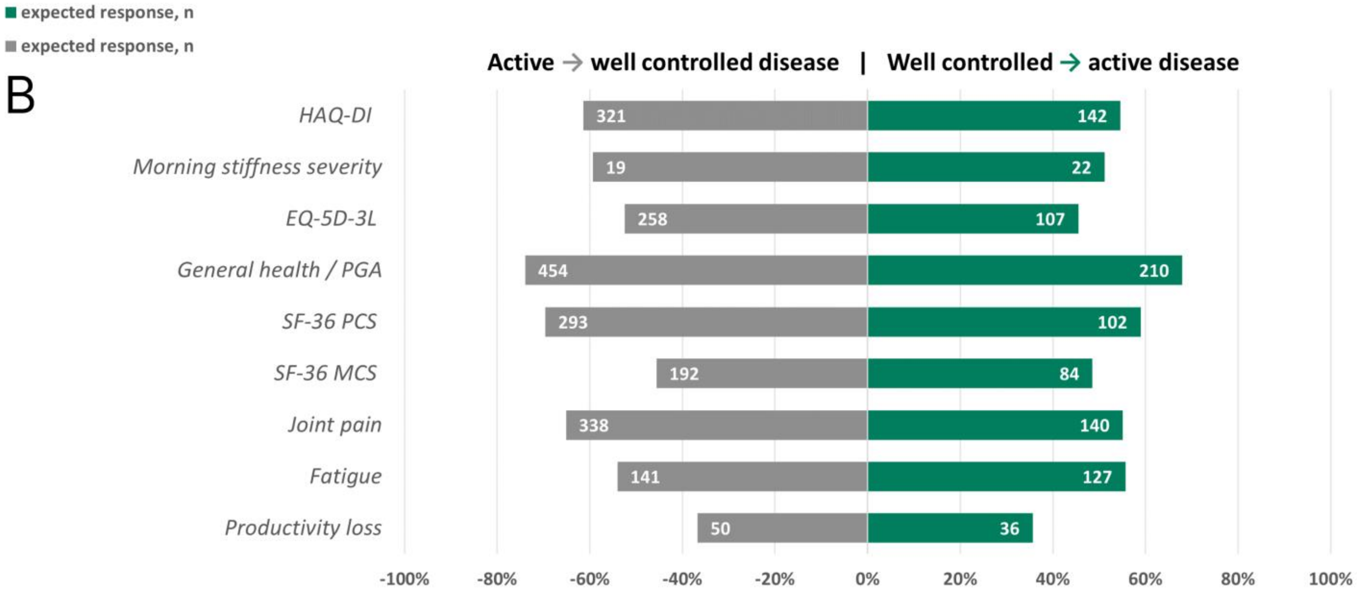

Supplementary figure S4 shows the proportion of RA-observations, early as well as established, with expected changes in PROM-score when changing from active (DAS>2.4) to well-controlled disease (DAS≤2.4) and vice versa. Figure A only includes observations for which the shift in disease status is accompanied with a minimal change in DAS of >0.6. Figure B shows results of disease status alterations calculated with the 3-item DAS as disease activity measure.

Abbreviations: EQ-5D-3L, 3-level EQ-5D; HAQ-DI, Health Assessment Questionnaire Disability Index; MCS, mental component score; PCS, physical component score; PGA, patient global assessment; and SF-36, RAND 36-item short form health survey.

References

1. Stucki G, Liang MH, Stucki S, Brühlmann P, Michel BA. A self-administered rheumatoid arthritis disease activity index (RADAI) for epidemiologic research. Arthritis & Rheumatism. 1995;38(6):795-8.

2. Nicklin J, Cramp F, Kirwan J, Greenwood R, Urban M, Hewlett S. Measuring fatigue in rheumatoid arthritis: A cross-sectional study to evaluate the Bristol Rheumatoid Arthritis Fatigue Multi-Dimensional questionnaire, visual analog scales, and numerical rating scales. Arthritis Care & Research. 2010;62(11):1559-68.

3. van Roijen L, Essink-Bot ML, Koopmanschap MA, Bonsel G, Rutten FF. Labor and health status in economic evaluation of health care. The Health and Labor Questionnaire. Int J Technol Assess Health Care. 1996;12(3):405-15.

4. Bouwmans C, Krol M, Severens H, Koopmanschap M, Brouwer W, Hakkaart-van Roijen L. The iMTA Productivity Cost Questionnaire: A Standardized Instrument for Measuring and Valuing Health-Related Productivity Losses. Value Health. 2015;18(6):753-8.

5. Middel B, Van Sonderen E. Statistical significant change versus relevant or important change in (quasi) experimental design: some conceptual and methodological problems in estimating magnitude of intervention-related change in health services research. International Journal of Integrated Care. 2002;2(4).
